# Physical and psychosocial benefits of yoga in cancer patients and survivors, a systematic review and meta-analysis of randomized controlled trials

**DOI:** 10.1186/1471-2407-12-559

**Published:** 2012-11-27

**Authors:** Laurien M Buffart, Jannique GZ van Uffelen, Ingrid I Riphagen, Johannes Brug, Willem van Mechelen, Wendy J Brown, Mai JM Chinapaw

**Affiliations:** 1 EMGO Institute for Health and Care Research, Department of Epidemiology and Biostatistics, VU University Medical Center, Van der Boechorststraat 7, Amsterdam, 1081 BT, The Netherlands; 2 Institute of Sport, Exercise and Active Living, Victoria University, Victoria, Australia; 3 School of Human Movement Studies, The University of Queensland, Queensland, Australia; 4 Faculty of Medicine, Norwegian University of Science and Technology, Trondheim, Norway; 5 EMGO Institute for Health and Care Research, Department of Public and Occupational Health, VU University Medical Center, Amsterdam, The Netherlands

**Keywords:** Yoga, Randomized controlled trial, Physical function, Psychosocial function, Quality of life, Cancer

## Abstract

**Background:**

This study aimed to systematically review the evidence from randomized controlled trials (RCTs) and to conduct a meta-analysis of the effects of yoga on physical and psychosocial outcomes in cancer patients and survivors.

**Methods:**

A systematic literature search in ten databases was conducted in November 2011. Studies were included if they had an RCT design, focused on cancer patients or survivors, included physical postures in the yoga program, compared yoga with a non-exercise or waitlist control group, and evaluated physical and/or psychosocial outcomes. Two researchers independently rated the quality of the included RCTs, and high quality was defined as >50% of the total possible score. Effect sizes (Cohen’s *d*) were calculated for outcomes studied in more than three studies among patients with breast cancer using means and standard deviations of post-test scores of the intervention and control groups.

**Results:**

Sixteen publications of 13 RCTs met the inclusion criteria, of which one included patients with lymphomas and the others focused on patients with breast cancer. The median quality score was 67% (range: 22–89%). The included studies evaluated 23 physical and 20 psychosocial outcomes. Of the outcomes studied in more than three studies among patients with breast cancer, we found large reductions in distress, anxiety, and depression (*d* = −0.69 to −0.75), moderate reductions in fatigue (*d* = −0.51), moderate increases in general quality of life, emotional function and social function (d = 0.33 to 0.49), and a small increase in functional well-being (d = 0.31). Effects on physical function and sleep were small and not significant.

**Conclusion:**

Yoga appeared to be a feasible intervention and beneficial effects on several physical and psychosocial symptoms were reported. In patients with breast cancer, effect size on functional well-being was small, and they were moderate to large for psychosocial outcomes.

## Background

Cancer represents a major public health concern. In Western countries, approximately one in three persons will be directly affected by cancer before the age of 75 years, with breast cancer, melanoma, colorectal cancer and prostate cancer comprising the most common types [1,2]. Due to medical advances, survival rates have improved over the past decade. For example, currently, the 5-year survival rates across all cancers are approximately 56% for male and 62% for female patients in Australia [1] and 58% and 64%, respectively, in the Netherlands [2]. However, cancer and its treatment are often associated with prolonged adverse physical and psychosocial symptoms, including reduced physical function and fitness and increased risk of anxiety, depression, and fatigue [3,4]. This greatly impacts the patient’s quality of life (QoL) [5,6]. Therefore, there is a need for effective methods to manage physical and psychosocial symptoms and to improve QoL of cancer patients and survivors.

Psychosocial interventions such as counselling, support groups and cognitive behavioural therapies may help patients cope with cancer and the psychosocial problems associated with cancer and cancer treatment, but are less likely to help with common physical issues such as loss of strength and flexibility, weight gain, and reduced physical function [7]. Findings from previous reviews and meta-analyses suggest that aerobic and resistance exercise attenuate a range of the physical problems associated with cancer and cancer treatment [3,4,6,8-16]. The benefits of these types of exercise include not only improved physical function, but also reduced fatigue and improved QoL. Unfortunately, many cancer patients perceive various barriers to exercise [17-21]. The most common physical barriers are physical discomfort and feeling sick. Psychosocial barriers include having low mood, feelings of self-consciousness relating to appearance and body image, fatigue and fear for overdoing it [20,22,23]. Because of these barriers, approximately one out of three adult cancer patients turns to complementary and alternative medicine techniques, mindfulness, or yoga, to help manage their symptoms [24-26].

Yoga is a ‘mind-body’ exercise, a combination of physical poses with breathing and meditation [27]. Several studies in the non-cancer population reported positive effects of yoga on physical outcomes including perceptual and motor skills [28], cardiopulmonary function [29], fitness [30], muscle strength, flexibility, stiffness, and joint pain [31-33]. Furthermore, a recent review of 10 studies comparing the effects of yoga asanas (postures) with those of ‘regular’ exercise, indicated that yoga may be as effective as exercise for improving health outcomes such as blood glucose and lipids, fatigue, pain, and sleep in healthy people and in people with conditions such as diabetes and multiple sclerosis [34].

Previous reviews [35,36] and a meta-analysis [37] of intervention studies have reported that yoga is feasible for patients with cancer, with improved sleep, QoL, mood and levels of stress. The current study extends previous work by our exclusive focus on 1) randomised controlled trials (RCTs), the most rigorous intervention study design; 2) yoga interventions that included physical postures and were not part of a larger program such as Mindfulness-Based Stress Reduction; and 3) a focus on both physical and psychosocial outcomes.

The aim of the present study is to conduct a systematic review and meta-analysis of the effects of yoga in cancer patients and survivors, focusing on both physical and psychosocial outcomes.

## Methods

### Literature search

IR, medical librarian, conducted the literature search in ten databases: AgeLine and AMED (Allied and Complementary Medicine Database), British Nursing Index, CINAHL, CENTRAL (The Cochrane Central Register of Controlled Trials), EMBASE, PEDro, PsycINFO, PubMed and SPORTDiscus (earliest to November 2011). In order to identify all relevant papers, a search was conducted with both thesaurus terms and free terms for ‘yoga’ in combination with an extensive list of search terms to identify intervention studies. RCTs were identified using search terms for certain publication types (e.g. randomized controlled trial and controlled clinical trial in PubMed) in combination with a list of free text terms in title and abstracts that could be used to describe RCTs (e.g. randomi*ed, randomly, trial, groups). Detailed search profiles are available on request from IR. Additional articles were identified by manually checking the reference list of included papers.

### Study inclusion criteria

Study inclusion criteria were: (i) design: RCT; (ii) population: adults with any cancer diagnosis either during or post treatment; (iii) intervention: yoga including physical postures (asanas); (iv) control group: non-exercise or wait-list; (v) outcome: physical and psychosocial outcomes. Only full-text articles written in English were included. Studies that included yoga as part of a larger intervention program (e.g., Mindfulness-Based Stress Reduction, meditation, or pranayama (breathing control) only) were excluded.

### Selection process and quality assessment

Titles and abstracts of the references were reviewed to exclude articles out of scope (JvU). Full-text articles of potentially relevant records were assessed for eligibility by two independent reviewers (LB and JvU).

LB and JvU independently assessed the quality of the included papers using a Delphi list developed by Verhagen et al. [38], which consists of nine equally weighted quality criteria to assess different methodological aspects (see below). This list has previously been used for the evaluation of methodological quality in systematic reviews of exercise programs [39-41]. Criteria have a ‘yes’ (=1), ‘no’ (=0) or ‘don’t know’ (=0) answer format. Disagreements between the reviewers were discussed and resolved, and in case of doubt, a third reviewer (MC) was consulted. Authors were contacted for additional information if it was not possible to score an item based on the information provided in the paper. Items scoring a “yes” contribute to the quality scores, ranging from 0 to 9 points. Where outcomes were assessed by self-report only, criterion 5 (blinding of the outcome assessor) was not applicable, and studies could obtain a maximum quality score of 8 points. A study was classified as a low quality study if the quality score was lower than 50% of the maximum possible score [41].

Criteria considered for quality assessment according to Verhagen et al. [38]

1. Was a method of randomization performed?

2. Was the treatment allocation concealed?

3. Were the groups similar at baseline?

4. Were the eligibility criteria specified?

5. Was the outcome assessor blinded?

6. Was the yoga instructor blinded (i.e. unaware of the study aim)?

7. Was the participant blinded?

8. Were point estimates and measures of variability (between groups comparison) presented for the primary outcomes?

9. Did the analysis include an intention-to-treat analysis?

### Data extraction

The following data were extracted by LB: (i) study population; (ii) type, intensity, frequency and duration of intervention, (iii) control group; (iv) outcome measures; and (v) effects on physical and/or psychosocial outcomes.

### Meta-analysis

Effect sizes were calculated (standardized mean difference *d*) for all individual studies by subtracting the average post-test score of the control group (M*c*) from that of the yoga intervention group (M*y*) and dividing the result by the pooled standard deviations of the yoga intervention group and the control groups (SD*yc*) [42]. An effect size of 0.5 thus indicates that the mean of the experimental group is half a standard deviation larger than the mean of the control group. Effect sizes of 0.56 to 1.2 are large, while effect sizes of 0.33 to 0.55 are moderate and effect sizes of 0 to 0.32 are small [43].

For outcomes that were investigated in >3 studies, individual effect sizes were pooled in Comprehensive Meta-Analysis (CMA; version 2.2.046). Because only one study did not include patients with breast cancer [44], the meta-analyses was conducted on data from studies including patients with breast cancer only. As we expected considerable heterogeneity, we calculated pooled effect sizes with the random effects model. This model assumes that the included studies are drawn from ‘populations’ of studies that differ from each other systematically (heterogeneity). In this model, the prevalence resulting from the included studies not only differs because of the random error within studies (fixed effects model), but also because of true variation in prevalence from one study to the next. We first tested the heterogeneity under the fixed model using the statistics I^2^ and Q. I^2^ describes the variance between studies as a proportion of the total variance. A value of 0% indicates no observed heterogeneity, and larger values show increasing heterogeneity, with 25% as low, 50% as moderate, and 75% as high heterogeneity [45]. When P values of the Q are above 0.05, the total variance is due to variance within studies and not to variance between studies. We ran the analyses on all studies and with outliers excluded. Studies with extreme values of which the 95% confidence interval had no overlap with the 95% confidence interval of the pooled estimate were considered as outliers.

## Results

**Figure 1:**
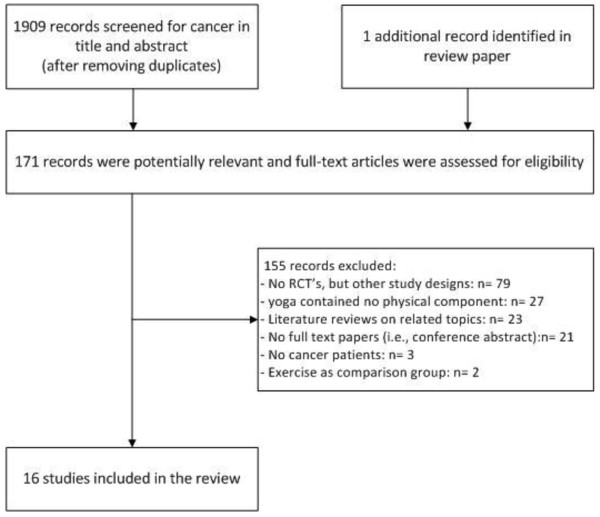
Flow chart.

After removing duplicates, the literature searches yielded a total of 1909 unique records. For 171 potentially relevant records, we checked full text (Figure 1). The majority of the studies (n = 79) were excluded because they were not designed as a RCT. Of the records identified in the database search, 15 records met the inclusion criteria. We found one additional RCT [31] from the reference list of the review by Smith and Pukall [35]. Both Vadiraja et al. [46-48] and Raghavendra et al. [49,50] published more than one paper on the same RCT, each describing different outcome measures and/or subpopulations. Thus 16 papers [31,32,44,46-58] of 13 RCTs were included in this systematic review. Details of the populations, yoga interventions, and outcomes of the included studies are presented in Tables 1, 2 and 3.

**Table 1:** Description of study populations in alphabetical order of first author

**Author, year**	**Diagnosis; treatment**	**number of participants (n); gender (%women); mean age (sd) and/or range**	**eligibility criteria**
Banasik, 2011 [56]	Breast cancer, (>2 mo) post-treatment	n = 18 (9Y, 9C) % women: 100% Age: 62.9 (7.1) years	**Inclusion** women with stages II-IV breast cancer at least 2 months post-treatment.
			**Exclusion** receiving Herceptin therapy, pregnant or lactating, had past or current history of other neoplasm, active serious infection or immune deficiency; history of psychiatric disorders or alcohol or drug abuse; steroid therapy or physical condition preventing yoga.
Banerjee, 2007 [51]	Breast cancer, during radiotherapy	n = 58 (35Y, 23C) % women: 100% Age: 44 (1.3) years	**Inclusion** Recently operated breast cancer, age between 30 and 70 years, Zubrod’s performance status 0–2 (ambulatory >50% of the time), high school education, treatment plan of radiotherapy or both radiotherapy and chemotherapy, consent to participate in the study.
			**Exclusion** Having any concurrent medical condition likely to interfere with the treatment; major psychiatric, neurological illness, or autoimmune disorders; cardiovascular illness; any known metastases. No exposure to other mutagens, smoking or alcohol for at least 3 months prior to pre-radiation blood donation.
Blank, 2003 [31]	Breast cancer stage I-III receiving antiestrogen or aromatase inhibitor hormonal therapy	N = 18 (9Y, 9C) % women: 100% Age: 48 – 69 years	**Inclusion** minimum of eight weeks post chemotherapy, estrogen receptor positive status, surgery for lumpectomy, modified mastectomy or full mastectomy (with/without reconstruction), a life expectancy greater than six months, adequate blood cell counts and kidney, liver, and cardiac function, physical and mental ability to attend all the Yoga training sessions.
			**Exclusion** women on Herceptin therapy, current steroid therapy, or other known immunomodulating medications, pregnancy or current lactation, a past or current history of another neoplasm, active serious infection or immune deficiency, documented alcohol or drug abuse, history of psychiatric disorders requiring use of psychotropic medication.
Bower, 2012 [58]	Breast cancer state 0 – II, at least 6 months after adjuvant cancer therapy.	n = 31 (16Y, 15C) % women: 100% Age: 54.4 (5.7) years	**Inclusion** originally diagnosed with stage 0 to II breast cancer; completed local and/or adjuvant cancer therapy (with the exception of hormone therapy) at least 6 months previously; ages 40 to 65 years; postmenopausal; no other cancer in last 5 years; experiencing persistent cancer-related fatigue.
			**Exclusion** chronic medical conditions or regular use of medications associated with fatigue; evidence that fatigue was driven primarily by a medical or psychiatric disorder other than cancer; evidence that fatigue was driven primarily by other noncancer-related factors; physical problems or conditions that could make yoga unsafe; a body mass index (BMI) >31 kg/m2.
Carson, 2009 [32]	Breast cancer; no current treatment (4.9 ± 2.4 years since diagnose)	n = 37 (17Y, 20C) % women: 100% Age: 54.4 (7.5) years	**Inclusion** Experiencing at least one hot flash per day on 4 or more days per week; no signs of active breast cancer; no current cytotoxic chemotherapy; diagnosed with breast cancer at stages IA-IIB ≥ 2 years before; no hormone replacement therapy currently or within prior 3 months; stabilized on constant regime of menopausal symptom medications and supplements for at least 3 weeks; if taking antidepressants, stabilized at a fixed dose for at least 3 months.
			**Exclusion** resided ≥ 70 miles from research site; unavailable to attend the intervention on the day and at the time offered; currently engaged in intensive yoga practice (> 3 days/week); having received treatment for serious psychiatric disorders (e.g. schizophrenia) in the previous 6 months; not English speaking
Chandwani, 2010 [55]	Breast cancer, during radiotherapy	N = 61 % women: 100% Age: 51.4 (8.0) range 37–68 years	**Inclusion** Women with stage 0-III breast cancer; ≥ 18 years; able to read, write and speak English; scheduled to undergo radiotherapy.
			**Exclusion** Patients who had any major psychiatric diagnosis or physical limitations that would prohibit participation in the yoga program.
Cohen, 2004 [44]	Lymphoma (18% Hodgkin), 61,5% active treatment	n = 39 % women: 61.5% Age: 51 years	**Inclusion** Patients with lymphoma who were either receiving chemotherapy or had received it within the past 12 months; ≥ 18 years; able to read and speak English.
			**Exclusion** Patients with major psychotic illnesses.
Culos-Reed, 2006 [52]	Breast cancer (85%); no current treatment (> 3 mo post-treatment)	n = 38 % women: 95% Age: 51.2 (10.3) years	**Inclusion** Cancer survivors who were currently not undergoing active treatment; no additional health concerns; ≥ 18 years; minimum 3 months post-treatment.
Danhauer, 2009 [53]	Breast cancer; 34% actively undergoing treatment	n = 44 % women: 100% Age: 55.8 (9.9) years	**Inclusion** Women ≥ 18 years; diagnosed with breast cancer; 2 to 24 months post-primary treatment (surgery) following initial diagnosis and/or had a recurrence of breast cancer within the past 24 months (regardless of treatment status); physically able to attend restorative yoga; able to understand English; free of medical contraindications reported by their physician.
Littman, 2011 [57]	Breast cancer; > 3 mo post-treatment	n = 63 % women: 100% Age: 60 (7.9) years	**Inclusion** Age between 21 and 75 years; completion of breast cancer treatment (stage 0-III) at least 3 months prior, BMI ≥24 kg/m^2^ (or ≥23 kg/m^2^ if of Asian descent).
			**Exclusion** Myocardial infarction or stroke in the previous 6 months, diabetes, current yoga practice, pregnancy or plans to become pregnant, factors that might lead to poor retention and yoga practice.
Moadel, 2007 [54]	Breast cancer; 48% medical treatment	n = 128 % women: 100% Age: 54.8 (9.9) range 28–75 years	**Inclusion** Age ≥ 18 years; new/recurrent breast cancer (stages I-III) diagnosis within previous 5 years; high performance status (Eastern Cooperative Oncology Group performance status of < 3); ability to speak English or Spanish; not actively practicing yoga.
Raghavendra, 2007 [49]	Breast cancer, during chemotherapy	n = 62 % women: 100% Age: n = 33 < 50 yrs; n = 29 > 50 yrs	**Inclusion** Recently diagnosed with operable breast cancer; aged between 30 and 70 years; Zubrod’s performance status 0–2; high school education; having a treatment plan with surgery followed by adjuvant chemotherapy or by both adjuvant radiotherapy and chemotherapy; consenting to participate in the study.
			**Exclusion** history of intestinal obstruction and any known sensitivity to any class of antiemetics.
Rao, 2009 [50]	Breast cancer, during adjuvant chemotherapy and radiotherapy	n = 98; % women: 100% Age: ?	**Inclusion** Recently diagnosed with operable breast cancer; aged between 30 and 70 years; Zubrod’s performance status 0–2; high school education; having a treatment plan with surgery followed by adjuvant radiotherapy and chemotherapy; consenting to participate in the study.
			**Exclusion** Having a concurrent medical condition likely to interfere with the treatment; any major psychiatric, neurological illness or autoimmune disorders; secondary malignancy.
Vadiraja, 2009 [46-48]	Breast cancer (stage II and III), during adjuvant	n = 88; % women: 100% Age: 46 (9.1) yrs yoga; 48.4 (10.2) yrs C.	**Inclusion** Recently diagnosed with operable breast cancer; aged between 30 and 70 years; Zubrod’s performance status 0–2; high school education; having a treatment plan with surgery followed by adjuvant chemotherapy or by both adjuvant radiotherapy and chemotherapy; consenting to participate in the study.
			**Exclusion** Having a concurrent medical condition likely to interfere with the treatment; any major psychiatric, neurological illness or autoimmune disorders; any known metastases; prescribed concurrent chemotherapy cycles during radiotherapy.

**Table 2:** Description of yoga programs, in alphabetical order of first author and attendance to yoga class

**Author, year**	**Yoga program (Y); Duration and frequency (D); Home practice (H) vs comparison (C)**	**Attendance**
Banasik 2011 [56]	**Y** Iyengar yoga given by expert Iyenger instructors, with focus on training and accepting the physical form of the body without specific meditation component.	Average 14 classes out of 16 (87.5%), range 12 – 15.
	**D** 8 weeks, twice a week, 90 min per session	
	**H -**	
	**C** wait-list	
Banerjee, 2007 [51]	**Y** Meditative practice, slow stretching and loosening exercises, motivation and counseling, yoga asanas, group awareness practices, pranayama, deep relaxation (yoga nidra) given by expert yoga trainers.	?
	**D** 6-weeks; 90 min per session	
	**H** Patients were provided with audio and video tools to practice at home and were followed up via telephone during weekends to ensure continuity of the practice.	
	**C** Supportive counseling and advised to take light exercise.	
Blank, 2003 [31]	**Y** Iyengar Yoga, including seated meditation, active asana, restorative poses, savasana.	?
	**D** 8 weeks, 2 times per week	
	**H** 1 home practice per week	
	**C** wait-list control	
Bower, 2012 [58]	**Y** Iyengar yoga classes were taught by a certified Junior Intermediate Iyengar yoga instructor and an assistant under the guidance of a senior teacher.	The mean number of yoga classes attended was 18.9 of 24 classes (78%), and the median was 22 of 24 classes (92%).
	**D** 12 weeks, twice a week, 90 min.	
	**H -**	
	**C** Health education classes were conducted for 120 min once a week for 12 weeks. Classes were led by a PhD-level psychologist with clinical experience in the treatment of breast cancer survivors.	
Carson, 2009 [32]	**Y** Yoga of Awareness given by certified yoga teacher: 40 min yoga poses, 10 min breathing techniques, 25 min meditation, 20 min of study pertinent topics and 25 min group discussion	Average 6 classes out of 8 (75%). 3 women less than 4 classes (3/17 = 17.6%)
	**D** 8 weeks, once a week, 120 min	
	**H** Patients were encouraged to practice daily at home with aid of CD recordings and illustrated hand books.	
	**C** Wait-list control	
Chandwani, 2010 [55]	**Y** The multidimensional yoga module was given a trained yoga instructor: 10 warm-up movements synchronized with breathing, 25 min maintenance in selected postures, 10 min deep relaxation, 5 min pranayama, 10 min mediation.	15 (50%) all 12 classes; 8 (28%) attended 11 classes; 1 (3%) attended 10 classes; 1 only 2 classes. One attended 3 classes, one 4, one 5, one 7 and one 8 classes. Average number of classes was 10.2 (85%); SD: 2.96; range 2 – 12. Home practice: 8 (28%) reported practicing
	**D** 6 weeks, 2 times per week; 60 min per session	
	**H** Patients were encouraged to practice type full yoga once per day outside the classes, supported by a 60-min audio CD of the yoga program and a manual with photographs and instructions.	
	**C** Wait-list control	
Cohen, 2004 [44]	**Y** Tibetan yoga sessions given by experienced instructor, divided into 4 aspects: controlled breathing and visualization, mindfulness, and postures.	32% all sessions; 26% 5 or 6 sessions; 32% 2 or 3 sessions; 10% 1 session
	**D** 7 weekly sessions	
	**H** Patients were encouraged to practice the techniques at least once per day, supported by audiotape that walked them though all of the techniques.	
	**C** Wait-list control	
Culos-Reed, 2006 [52]	**Y** Classes were led by a certified yoga instructed and included 10 min gentle breathing; 50 min Yoga asanas; 15 min savasana.	
	**D** 7 weeks, 75 min.	
	**H** -	
	**C** Wait-list control	
Danhauer, 2009 [53]	**Y** Restorative yoga classes were taught by a yoga instructor with cancer-specific yoga training and combined yoga asanas, pranayama, savasana.	Mean 5.8 (3.4) classes out of 10 (58%) 2 (10%) women 100%; 3 (14%) 0% of classes
	**D** 10 weekly 75-min classes	
	**H** -	
	**C** Wait-list control	
Littman, 2011 [57]	**Y** Viniyoga, a Hatha therapeutic type o f yoga given by certified experienced yoga instructors: 5–10 min centering exercises to promote relaxation and internal focus, 50–60 min of seated and standing poses, 10–15 min guided relaxation, breathing exercises and meditation.	Mean 19.6 (range 1–61; median 20.5) classes. Home practice: 55.8 times (range 2 – 102; median 62).
	**D** 6 months, 5 times per week including at least one 75-min class	
	**H** patients were given a DVD, VD and booklets of four home practices lasting 20–30 min each.	
	**C** Wait-list control	
Moadel, 2007 [54]	**Y** Classes were given by a certified yoga instructor and included 3 yoga components: physical stretches and poses, breathing exercises, and meditation.	High adherence (>6 classes): n = 33 (; Low adherence (1–6 classes), n = 24; No adherence (0 classes), n = 27. Average attendance 7 out of 12 classes (58%).
	**D** 12 weekly 1.5 hrs classes (more allowed)	
	**H** Patients were asked to practice yoga at home daily and given an audiotape/compact disk for guidance.	
	**C** Wait-list control	
Raghavendra, 2007 [49]	**Y** Integrated yoga program administered by an instructor: asanas, breathing exercise, pranayama, meditation and yogic relaxation techniques with imagery.	?
	**D** 30 min before the start of the chemotherapy infusion (once in 10 days, number of cycles 4–8).	
	**H** Patients were provided with audiotapes of these exercises for home practice and asked to practice daily for 1 h for 6 days/week during intervals between chemotherapy cycles.	
	**C** Supportive therapy and coping preparation	
Rao, 2009 [50]	**Y** Integrated yoga program administered by an instructor: asanas, breathing, pranayama, mediation and yogic relaxation techniques with imagery.	?
	**D** Four sessions during pre- and post operative period, 3 in-person sessions per week for 6 weeks during radiotherapy. During chemotherapy, subjects underwent person sessions during their hospital visits for chemotherapy administration (once in 21 days) and an additional yoga session once in 10 days.	
	**H** Patients were given booklets, audiotapes with instructions on practices for home practice.	
	**C** Supportive therapy sessions	
Vadiraja, 2009 [46-48]	**Y** Integrated yoga program administered by an instructor: asanas, breathing, pranayama, mediation and yogic relaxation techniques with imagery.	29.7% attended 10-20% supervised sessions, 56.7% attended 20–25, 13.7% attended >25 supervised sessions over a 6-week period. Attend minimal 3x/wk for 6 weeks → 18 classes.
	**D** Minimum of 3 in-person sessions per week for 6 weeks during radio treatment; 1 hour per session. In total between 18–24 yoga sessions.	
	**H** Patients were given booklets, audiotapes with instructions on practices for home practice.	
	**C** Supportive therapy with education. 15-min counseling sessions once every 10 days during 6 weeks (3 or 4 sessions in total).	

Asana = physical posture; Pranayama = breathing practice, voluntary regulated nostril breathing; Yoga nidra = deep relaxation; Savasana = the corpse pose, relaxation.

**Table 3:** Description of physical and psychosocial outcomes and between group differences (yoga vs control), in alphabetical order of first author

**Author, year**	**Physical outcomes**	***Between group difference***	**Psychosocial outcomes**	***Between group difference***
Banasik, 2011 [56]	FACT		FACT	
	- Physical well-being	N.S.	- emotional well-being	N.S.
	- Functional well-being	N.S.	- social well-being	N.S.
	Cortisol, morning	N.S.	Breast cancer concerns	N.S.
	Cortison, noon	P = 0.004	Fatigue	P = 0.003
	Cortisol, 5 p.m.	P = 0.004		
	Cortisol, 10 p.m.	N.S.		
Banerjee, 2007 [51]	DNA damage	14,5% less DNA damage in Yoga group; p < 0.001	Anxiety (HADS-A)	48% reduction in yoga group vs 28% increase in controls; p < 0.001
			Depression (HADS-D)	57.5% decrease in yoga vs 24% decrease in controls; p < 0.001
			Perceived stress (PSS)	26.9% reduction in yoga vs 7% increase in controls; p < 0.001
Blank, 2003 [31]	25% had relieved joint aches and shoulder stiffness	NA	100% perceived direct stress reduction	NA
			88% felt more relaxed in daily life, more aware of body posture, improved body image	NA
			63% had improved mood and less anxiety	NA
Bower, 2012 [58]	Lower extremity strength and endurance (timed chair stands)	1.31 (−5.00; 2.38, N.S.	Fatigue (FSI)	−1.24 (−0.04; -2.45), p < 0.05
	Flexibility (functional reach test)	−2.00 (5.76; -9.98), N.S.	Vigor	4.80 (1.86; 7.74), p < 0.05
			Depression (BDI)	−5.80 (−1.74; -9.86), p < 0.05
			Sleep quality (PSQI)	0.20 (2.78; -2.38), N.S.
			Perceived stress (PSS)	−1.77 (1.71; -5.26), N.S.
Carson, 2009 [32]	Hot flash frequency	P = 0.0017	Negative mood	P = 0.099
	Hot flash severity	P = 0.0019	Relaxation	P = 0.543
	Hot flash total	P < 0.0001	Vigor	P = 0.005
	Joint pain	P < 0.0001	Acceptance	P = 0.058
	Night sweats	N.S.	Symptom-related bother	P < 0.0001
			Fatigue	P = 0.001
			Sleep disturbance	P = 0.007
Chandwani, 2010 [55]	SF-36		SF-36	
	- Physical component summary	ES = 0.44; P = 0.04	- Mental component summary	N.S.
	- Physical function	ES = 0.46; p = 0.04	- Mental health	N.S.
	- body pain	N.S.	- Role physical	N.S.
			- Role emotional	N.S.
			- Social function	N.S.
			- vitality	N.S.
			- General HRQoL	ES = 0,47; p = 0.005
			Depression (CES-D)	N.S.
			Anxiety (STAI)	N.S.
			Distress (IES)	
			- Intrusion	N.S.
			- Avoidance	N.S.
			Fatigue (BFI)	N.S.
			Sleep (PSQI)	N.S.
			Benefit finding (BFS)	N.S.
Cohen, 2004 [44]			Distress (IES)	N.S.
			Anxiety (STAI)	N.S.
			Depression (CES-D)	N.S.
			Sleep disturbances (PSQI)	
			- Total score	P = 0.004
			- Sleep quality	P = 0.02
			- Sleep latency	P = 0.01
			- Sleep duration	P = 0.03
			- Sleep efficiency	N.S.
			- Sleep medications	P = 0.02
			- Daytime dysfunction	N.S.
			Fatigue (BFI)	N.S.
Culos-Reed, 2006 [52]	Physical activity (LSI)	N.S.	Mood (POMS)	
	Weight	N.S.	- Total mood	P < 0.10
	Systolic and diastolic blood pressure	N.S.	- Tension-anxiety	P < 0.10
	Hand grip strength	N.S.	- Depression-dejection	P < 0.10
	Distance walked	N.S.	- Confusion-bewilderment	P < 0.10
	Perceived exertion	N.S.	- Vigor	N.S.
	Flexibility (sit and reach)	N.S.	- Anger-hostility	N.S.
	EORTC-QLQ-C30		Symptoms of stress (SOSI)	
	- Physical function	N.S.	- Peripheral manifestations	N.S.
	- pain	N.S.	- Cardiopulmonary symptoms	N.S.
	- nausea and vomiting	N.S.	- Symptoms of arousal	N.S.
	- dyspnea	P < 0.05	- Upper respiratory symptoms	N.S.
	- appetite	N.S.	- Central neurological symptoms	N.S.
	- constipation	N.S.	- Gastrointestinal symptoms	P < 0.10
	- diarrhea	P < 0.05	- Muscle tension	N.S.
			- Habitual patterns	N.S.
			- Depression	N.S.
			- Anxiety/fear	N.S.
			- Emotional irritability	P < 0.10
			- Cognitive disorganization	P < 0.10
			HRQoL (EORTC QLQ-C30)	
			- global quality of life	P < 0.01
			- emotional function	P < 0.05
			- cognitive function	N.S.
			- social function	N.S.
			- role function	N.S.
			- fatigue (POMS)	N.S.
			- sleep disturbance	N.S.
Danhauer, 2009 [53]	Physical function (SF-12)	N.S.	Mental health (SF-12)	P = 0.004
	FACT		Depression (CES-D)	P = 0.026
	- Physical well-being	N.S.	Fatigue (FACT-fatigue)	N.S.
	- Functional well-being	N.S.	Negative affect (PANAS-NA)	P = 0.014
			Positive affect (PANAS-PA)	P = 0.01
			FACT-General	P = 0.052
			- Social well-being	N.S.
			- Emotional well-being	P = 0.042
			Spiritual well being (FACIT Sp)	
			- peace/meaning	P = 0.0009
			- role of faith	N.S.
			Sleep disturbances (PSQI)	
			- Total score	N.S.
			- Sleep quality	N.S.
			- Sleep latency	P = 0.078
			- Sleep duration	N.S.
			- Sleep efficiency	N.S.
			- Sleep medications	P = 0.10
			- Daytime dysfunction	N.S.
Littman, 2011 [57]	FACT		Overall QoL (FACT-G)	N.S.
	- Physical well-being	N.S.	Breast-cancer subscale	N.S.
	- Functional well-being	N.S.	- Social well-being	N.S.
	Physical Activity (MAQ)	N.S.	- Emotional well-being	N.S.
	BMI	N.S.	- social/family well-being	N.S.
	Waist circumference	−3.1 (−5.7; -0.4)	Fatigue (FACIT-F)	N.S.
	Hip circumference	N.S.		
	weight	N.S.		
Moadel, 2007 [54]	FACT		Overall QoL (FACT-G)	P < 0.01†
	- Physical well-being	N.S.	- Social well-being	ES = −0.22 (−3.78 to −0.36); P = 0.018
	- Functional well-being	N.S.	- Emotional well-being	P = 0.018*; P < 0.05†
			Fatigue (FACT-fatigue)	N.S.
			Spiritual well-being (FACIT Sp)	P = 0.009†
			Distressed Mood (DMI)	P < 0.05†
			- Anxious/sad	P = 0.046†
			- Irritability	P = 0.0275†
			- Confusion	N.S.
Raghavendra, 2007 [49]	Nausea frequency	P = 0.01	Anxiety (STAI)	P < 0.001
	Nausea severity	P < 0.01	Depression (DBI)	P < 0.001
	Vomiting frequency	P = 0.06	Number of distressful symptoms	P = 0.002
	Vomiting severity	P = 0.05	Severity of symptoms	P < 0.001
	Total toxicity score	P < 0.001	Symptom distress	P < 0.001
			Overall quality of life (FLIC)	P < 0.001
Rao, 2009 [50]			State anxiety (STAI)	ES = 0.33; P < 0.05 (ITT)
			Trait anxiety (STAI)	ES = 0.24; NS (ITT)
			Symptom distress	P = 0.001
Vadiraja, 2009a [46]	Cortisol level at 6 am	ES = 0.24;P < 0.05	Anxiety (HADS-A)	ES = 0.31; P < 0.001
	Cortisol level at 9 am	N.S.	Depression (HADS-D)	ES = 0.31; P < 0.01
	Cortisol level at 9 pm	N.S.	perceived stress (PSS)	ES = 0.36; P < 0.001
	Mean pooled diurnal cortisol	ES = 0.27; P < 0.05		
Vadiraja, 2009b [47]	EORTC QLQ-C30		Positive Affect (PANAS)	ES = 0.59; P = 0.007
	- Physical function	ES = 0.16; N.S.	Negative Affect (PANAS)	ES = 0.84; P = 0.001
			HRQoL (EORTC QLQ-C30)	
			- Role function	ES = 0.19; N.S.
			- Emotional function	ES = 0.71; P = 0.001
			- Cognitive function	ES = 0.48; P = 0.03
			- Social function	ES = 0.21; N.S.
Vadiraja 2009c [48]	Physical distress (RSCL)	ES = 0.33; p = 0.02	Psychological distress (RCSL)	ES = 0.39; p < 0.001
	EORTC-QLQ-C30		EORTC QLQ-C30	
	- pain	ES = 0.14; N.S.	- fatigue	ES = 0.33; N.S.
	- nausea and vomiting	ES = 0.05; N.S.	- insomnia	ES = 0.47; N.S.
	- dyspnea	ES = 0.01; N.S.		
	- appetite loss	ES = 0.38; N.S.		
	- diarrhea	ES = 0.01; N.S.		
	- constipation	ES = 0.14; N.S.		
	Activity level	ES = 0.14; N.S.		

BDI = Beck’s Depression Inventory; BFI = Brief Fatigue Inventory; CES-D = Centers for Epidemiologic Studies-Depression; CT = chemotherapy; DMI = Distressed Mood Index; EORTC-QoL C30 = European Organization for the Research and Treatment of Cancer-Quality of Life; ES = effect size; FACT-G = Functional Assessment of Cancer Therapy-General; FACIT = Functional Assessment of Chronic Illness Therapy; FLIC = Functional Living Index for Cancer; FSI = Fatigue Symptom Inventory; HADS = Hospital Anxiety and Depression Scale; IES = Impact of Events Scale; ITT = Intention to treat; LSI = Leisure Score Index; MAQ = Modifiable Activity Questionnaire; NA = not assessed; N.S. = not significant; PANAS = Positive and Negative Effect Schedule; POMS = Profile of Mood states; PSQI = Pittsburgh Sleep Quality Index; PSS = perceived stress scale; RSCL = Rotterdam Symptom Check List; SOSI = Symptoms of Stress Inventory; STAI = Spielberger’s State Trait Anxiety Inventory.

### Quality assessment

Results of the methodological quality assessment are presented in Table 4. Median quality score was 67% (range 22–89%). All but one study [31] were of high quality. All included studies used randomization. In all but one [31] study treatment allocation was concealed, and groups were comparable at baseline, or dissimilarities at baseline were adequately adjusted for in the analyses. All studies adequately specified the eligibility criteria of the study population. The outcome assessor was blinded in five papers [32,51,52,57,58], but this criterion was not applicable in the seven papers using self-reported outcomes only [44,47,49,50,55]. In five papers [51,52,55-57], the yoga instructor was blinded as he or she was unaware of the study aim. Participants were blinded in two papers [51,58]; Banerjee [51] informed us that their study was double blinded. In four papers, point estimates and 95% confidence intervals (CI) for between group differences were reported [47,50,54,58]. One paper [44] reported 95% CI only, and three papers [46,48,55] only presented effect sizes, without 95% CI. In nine papers [32,47,48,50,52-55,58], data were analyzed on an intention-to-treat basis.

**Table 4:** Quality assessment sorted by study population and quality score

**First author, year**	**1**	**2**	**3**	**4**^**a**^	**5**	**6**	**7**	**8**	**9**	**score**	**%**
Banasik, 2011 [56]	Y	Y	Y	Y	SR	Y ^C^	N	N	N	5	63%
Banerjee, 2007 [51]	Y	Y ^C^	Y	Y	Y	Y ^C^	Y ^C^	N	N	7	78%
Blank, 2003 [31]	Y	?	?	Y	N	?	N	N	N	2	22%
Bower, 2012 [58]	Y	Y	Y	Y	Y	?	Y	Y	Y	8	89%
Carson, 2009 [32]	Y	Y	Y	Y	Y	N ^C^	N	N	Y	6	67%
Chandwani, 2010 [55]	Y	Y ^C^	Y	Y	SR	Y ^C^	N	N, ES no CI	Y	6	75%
Cohen, 2004 [44]	Y	Y	Y	Y	N^C^ (SR)	N ^C^	N	N, only 95% CI	N	4	50%
Culos-Reed, 2006 [52]	Y	Y	Y	Y	Y ^C^	Y ^C^	N	N	Y	7	78%
Danhauer, 2009 [53]	Y	Y	Y	Y	N^C^	N ^C^	N	N	Y	5	56%
Littman, 2011 [57]	Y	Y ^C^	Y	Y	Y ^C^	Y ^d^	N	Y	N	7	78%
Moadel, 2007 [54]	Y	Y ^C^	Y ^b^	Y	N^C^	N ^C^	N	Y	Y	6	67%
Raghavendra, 2007 [49]	Y	Y	Y	Y	SR	N	N	N	N	4	50%
Rao, 2009 [50]	Y	Y	Y	Y	SR	N	N	Y	Y	6	67%
Vadiraja, 2009a [46]	Y	Y	Y	Y	?	?	N	N, ES no CI	N	4	50%
Vadiraja, 2009b [47]	Y	Y	Y	Y	SR	?	N	Y	Y	6	75%
Vadiraja, 2009c [48]	Y	Y	Y	Y	SR	?	N	N, ES no CI	Y	5	56%

*NA* not applicable, *Y* yes, *N* no, *?* unclear, ^**a**^ If only exclusion criteria were reported, this was rated as ‘unclear’; ^b^ In the analyses, the baseline differences were included as covariates. ^C^ after contacting authors; ^d^ Yoga instructors were aware that the study aim was to determine the feasibility of conducting a yoga intervention in overweight and obese breast cancer survivors (not efficacy). *SR* self report, *CI* Confidence interval, *ES* effect size.

### Study population

Details of the study populations are reported in Table 1. Twelve studies included patients with breast cancer and one study focused on patients with lymphomas [44]. Five studies in patients with breast cancer studies took place during cancer treatment: three studies (five papers [46-48,51,55]) during radiotherapy, one study [31] during hormone therapy, and one study (two papers, [49,50]) during chemotherapy with or without additional radiotherapy. Five studies [32,52,56-58] focused on breast cancer survivors who had completed treatment, and two studies [53,54] included patients and survivors both during and after treatment. The study in patients with lymphomas included patients during and after active treatment [44]. Sample sizes ranged from 18 to 128 patients, with seven studies including less than 50 patients, and only one study with more than 100 patients. Average age of the participants ranged from 44 to 63 years. One study did not report the age of the patients [50]. Eleven studies in patient with breast cancer included women only, one study [52] in mainly breast cancer patients (85%) included 5% men, and the study in lymphoma patients [44] included 39% men.

### Yoga program

The content of the yoga programs is summarized in Table 2. All included a supervised yoga program with physical poses (yoga asanas), combined with breathing techniques (pranayama) and relaxation or meditation (savasana or dhanya).

All yoga classes were led by experienced yoga instructors. Median program duration was seven weeks with a range of six weeks to six months. In the study by Rao et al. [50], the program duration depended on the number of chemotherapy cycles, which ranged from four to eight. In this latter study, supervised sessions were conducted for 30 min before chemotherapy once every ten days. Furthermore, patients were provided with audiotapes of the exercises for home practice and asked to practice 1 h daily for 6 days/week during intervals between chemotherapy cycles [49]. In general, the number of classes per week ranged from one to three, and home practice was encouraged in nine studies, supported by audio or videotapes. Session duration ranged from 30 to 120 min; three studies did not report the session duration [31,44,50].

In nine studies [31,32,44,52-57] the yoga program was compared with a wait-list control group. In three studies [46-51], the control group received supportive therapy with education, counseling, or coping preparation. In one study, the control group received health education classes [58].

### Effects

Tables 5 and 6 present an overview of the effects of yoga on physical and psychosocial outcomes, respectively (for details, see Table 3). Fourteen papers reported on both physical and psychosocial outcomes, and two papers reported on psychosocial outcomes only.

**Table 5:** Summary of the effects of yoga compared to control on physical outcomes

**PHYSICAL**	**1**	**2**	**3**	**4**	**5**	**6**	**7**	**8**	**9**	**10**	**11**	**12**	**13**	**14**	**15**	**16**
Reference	[56]	[51]	[31]	[58]	[32]	[55]	[44]	[52]	[53]	[57]	[54]	[49]	[50]	[46]	[47]	[48]
Year	2011	2007	2003	2011	2009	2010	2004	2006	2009	2011	2007	2007	2009	2009a	2009b	2009c
Sample size	18	58	18	31	37	61	39	38	44	63	128	62	98	88	88	88
Treatment	AT	RT	HT	AT	AT	RT	Mix	AT	Mix	AT	mix	CT	CT + RT	RT	RT	RT
Quality	high	high	low	high	high	high	high	high	high	high	high	high	high	high	high	high
*Physical function*																
Physical function	N.S.					↑		N.S.	N.S.	N.S.	N.S.				N.S.	
Functional well being	N.S.								N.S.	N.S.	N.S.					
*Physical symptoms*																
Pain					↓	N.S.		N.S.								N.S.
Nausea vomiting								N.S.				↓				N.S.
Toxicity												↓				
Diarrhoea								↓								N.S.
Constipation								N.S.								N.S.
Appetite								N.S.								N.S.
Dyspnea								↓								N.S
Hot flashes					↓											
Night sweats					N.S.											
*Activity/fitness*																
Physical Activity								N.S.		N.S.						N.S.
Weight								N.S.		N.S.						
Body mass index										N.S.						
Waist circumference										↓						
Hip circumference										N.S.						
Flexibility				N.S.				N.S.								
Strength				N.S.				N.S.								
Fitness/distance walked								N.S.								
Perceived exertion								N.S.								
*Biological Variables*																
DNA Damage		↓														
Cortisol	↓													↓		
Blood pressure								N.S.								

↑ = increase after yoga compared to control; ↓ decrease after yoga compared to control; N.S. no significant differences between yoga and control

*AT* after treatment, *CT* chemotherapy, *HT*, hormonal therapy, *RT* radiotherapy, *mix* mixed group of patients during and after treatment.

**Table 6:** Summary of the effects of yoga compared to control on psychosocial outcomes

**PSYCHOSOCIAL**	**1**	**2**	**3**	**4**	**5**	**6**	**7**	**8**	**9**	**10**	**11**	**12**	**13**	**14**	**15**	**16**
Reference	[56]	[51]	[31]	[58]	[32]	[55]	[44]	[52]	[53]	[57]	[54]	[49]	[50]	[46]	[47]	[48]
Year	2011	2007	2003	2011	2009	2010	2004	2006	2009	2011	2007	2007	2009	2009a	2009b	2009c
Sample size	18	58	18	31	37	61	39	38	44	63	128	62	98	88	88	88
Treatment	AT	RT	HT	AT	AT	RT	Mix	AT	Mix	AT	Mix	CT	CT + RT	RT	RT	RT
Quality	high	high	low	high	high	high	High	high	high	high	high	high	high	high	high	high
Distress		↓	↓	N.S.	↓	N.S.	N.S.					↓	↓	↓		↓
Anxiety		↓	↓			N.S.	N.S.				↓	↓	↓	↓		
Depression		↓		↓		N.S.	N.S.		↓			↓		↓		N.S.
Fatigue	↓			↓	↓	N.S.	N.S.		N.S.	N.S.	N.S.					
Sleep disturbance				N.S.	↓	N.S.	↓		N.S.							↓
General HRQoL	↑					↑		↑	↑	N.S.	↑	↑				
Emotional function	N.S.					N.S.			↑	N.S.	↑				↑	
Social function	N.S.					N.S.		N.S.	N.S.	N.S.	↑				N.S.	
Role function						N.S.									N.S.	
Cognitive function															↑	
Positive affect									↑						↑	
Negative affect									↑						↑	
Vigor				↑	↑											
Mood			↑		↑											
Anger-hostility											↓					
Spirituality									↑		↑					
Relaxation			↑		↑											
Confusion											N.S.					
Mental Health						N.S.			↑							
Acceptance					↑											

↑ = increase after yoga compared to control; ↓ decrease after yoga compared to control; N.S. no significant differences between yoga and control

*AT* after treatment, *CT* chemotherapy, *HT* hormonal therapy, *RT* radiotherapy, *mix*, mixed group of patients during and after treatment.

#### Physical outcomes

Twenty-three physical outcomes were examined in thirteen of the included papers (Table 5). In addition to self-reported physical function and functional well-being, outcomes included nine physical symptoms (e.g., pain, nausea, and dyspnoea), nine measures of physical activity and fitness, and three biological variables. However, except for physical function, functional well being, and pain, the outcomes were studied in only three studies or less, thus we considered this evidence insufficient to draw conclusions on the effectiveness of yoga on these outcomes. After excluding an outlier [55], the pooled effect size of yoga on physical function in patients with breast cancer was small and insignificant (*d* = 0.17; 95% CI = −0.06 to 0.40), see Table 7. Further, in patients with breast cancer, yoga resulted in a small but significant increase in functional well-being (*d* = 0.31; 95% CI = 0.04 to 0.58). Pain was evaluated in four studies, of which standard deviations to calculate effect sizes were not available in two studies [32,44]. The average effect size of the other two studies among patients with breast cancer [48,55] was large (*d* = −0.64; 95% CI = −0.98 to −0.31).

**Table 7:** Pooled effects of yoga on physical and psychosocial outcomes in patients with breast cancer

**Outcome**	**# studies**	**Pooled effect**				**Test of heterogeneity**		
***Physical outcomes***		***d***	**95% CI**	**Z**	**P**	**I**^**2**^	**Q**	**P**
Physical function	6	0.60	−0.05 to 1.25	1.81	0.07	87.51	40.03	<0.0001
	5^a^	0.17	−0.06 to 0.40	1.48	0.14	0.00	1.20	0.88
Functional well-being	4	0.31	0.04 to 0.58	2.24	0.03	0.00	1.25	0.74
***Psychosocial outcomes***		***d***	**95% CI**	**Z**	**P**	**I**^**2**^	**Q**	**P**
Distress	7	−0.95	−1.49 to −0.49	−4.04	<0.001	80.79	31.24	<0.001
	6^b^	−0.75	−1.09 to −0.42	−4.39	<0.001	59.59	12.37	0.03
Anxiety	7	−1.25	−1.93 to −0.56	−3.64	<0.001	91.45	70.20	<0.001
	6^b^	−0.77	−1.08 to −0.46	−4.86	<0.001	58.42	12.03	0.03
Depression	7	−1.47	−2.42 to −0.53	−3.05	0.002	93.29	89.46	<0.001
	6^b^	−0.69	−1.02 to −0.37	−4.21	<0.001	42.15	8.64	0.12
Fatigue	7	−0.51	−0.79 to −0.22	−3.46	0.001	43.52	10.62	0.10
Sleep disturbance	4	−0.26	−0.53 to 0.02	−1.82	0.07	0.00	1.25	0.74
General HRQoL	7	0.88	0.25 to 1.50	2.75	0.006	86.49	44.41	<0.001
	6^a^	0.61	0.16 to 1.06	2.50	0.008	69.79	16.55	0.005
	5^a,c^	0.37	0.11 to 0.62	2.85	0.004	0.00	3.40	0.49
Emotional function	5	0.49	0.16 to 0.81	2.93	0.003	26.58	5.45	0.24
Social function	6	0.33	0.12 to 0.54	3.12	0.002	0.00	1.94	0.86

^a^ Excluding outlier Chandwani et al. 2010 [55]; ^b^ Excluding outlier Banerjee et al. [51]. ^c^ Excluding outlier Raghavendra et al. 2007 [49].

#### Psychosocial outcomes

Twenty psychosocial outcomes were examined in the fifteen included papers (Table 6). The effects of yoga on distress, anxiety, depression, fatigue, sleep, general QoL, emotional function and social function were evaluated in three or more studies. After excluding outliers, yoga resulted in significant large reductions in distress (*d* = −0.75; 95% CI = −1.09 to −0.42), anxiety (*d* = −0.77; 95% CI = −1.08 to −0.46), and depression (*d* = −0.69; 95% CI = −1.02 to −0.37), moderate reductions in fatigue (*d* = −0.51; 95% CI = −0.79 to −0.22), and moderate increases in general HRQoL (*d* = 0.37; 95% CI = 0.11 to 0.62), emotional function (*d* = 0.49; 95% CI = 0.16 to 0.81), and social function (*d* = 0.33; 95% CI = 0.12 to 0.54) in breast cancer patients, see Table 7. Effects on sleep disturbances were small and insignificant (*d* = −0.26; 95% CI = −0.53 to 0.02). In patients with lymphoma, however, Cohen et al. [44] found a significant reduction in sleep disturbances (*d* = −1.00; 95% CI = −3.8 to −0.8). Although some studies found beneficial effects on other psychosocial outcomes, including positive and negative effect, mood, spirituality and relaxation (Table 6), these were studied in less than three studies. Therefore, this evidence was considered to be insufficient.

### Dropout and attendance

Dropout from the studies, defined as the number of randomized participants without post-intervention measurement ranged from 0 to 38%. Attendance at the yoga classes was reported in nine studies [32,44,47,53-57], and varied between 58 and 88% (Table 2). Vadiraja et al. [47] reported that the level of adherence did not influence results on QoL, positive and negative affect [47]. Four other studies reported on the influence of intervention adherence on outcomes. Danhauer et al. [53] reported that better intervention adherence was associated with higher self-reported physical function and QoL. In contrast, Moadel et al. [54] found similar improvements in QoL among participants with low and high class attendance, but found a positive association between intervention attendance and improved mood. Carson et al. [32] also showed that greater mean yoga practice time was associated with less fatigue, less symptom bother, and more acceptance at post-treatment, and tended to be associated with less sleep disturbances. Littman et al. [57] reported that generally, the benefits were greater among women who attended more facility-based classes, but results were not entirely consistent.

### Safety

Five studies evaluated adverse events and provided this information in the manuscripts [47,50,53,57,58]. Four studies reported that there were no adverse events and one study [58] reported one adverse event of a participant with a history of back problems, who experienced a back spasm in yoga class. After evaluation by her physician, she was able to return to class and complete the intervention.

## Discussion

This review and meta-analysis described and evaluated sixteen papers examining yoga as an intervention to manage physical and psychosocial symptoms in cancer patients and survivors. In contrast to previous reviews [35,36] and meta-analysis [37] we only included studies focusing on yoga interventions with physical postures, and evaluating the effectiveness on physical and/or psychosocial outcomes. Yoga appeared to be a feasible intervention, and beneficial effects on several physical and psychosocial symptoms were reported, with a small effect on functional well-being and moderate to large effects on various psychosocial outcomes.

### Physical outcomes

Due to the limited number of studies per physical outcome, evidence for physical effects of yoga was generally insufficient to draw firm conclusions. The effects of yoga on physical function and functional well-being were small. This may be related to the short intervention duration; only two studies lasted 12 weeks or longer [54,57], all others were shorter, ranging from 6 to 10 weeks (median = 7). To improve physical function and fitness, longer intervention duration may be required. The lack of significant improvements in physical function and fitness may also be related to the relatively low intensity of certain types of yoga [52,59]. Nevertheless, in healthy older adults, a 6-month yoga intervention resulted in improved physical outcomes such as timed 1-leg stand, flexibility, and energy [60]. These beneficial effects may be related to the lower baseline cardiorespiratory fitness of older adults compared with younger patients, and the longer intervention duration in that specific study. Significant improvements in treadmill time and estimated peak oxygen uptake as a result of yoga have also been shown in a small group of patients with chronic heart failure [61]. A systematic review of studies comparing yoga with other forms of exercise concluded that in both healthy people and in patients with chronic diseases, yoga may be as effective or better than other forms of exercise at improving a variety of health-related outcome measures, including physical outcomes such as muscle strength and flexibility [34]. One study with healthy sedentary elderly people has reported that peak oxygen uptake increased by 11% after yoga, compared with 24% after aerobic training [62]. Although patients perceived that they had improved fitness after 12 weeks of yoga [63], future empirical evidence should indicate whether yoga is as beneficial as endurance or strength exercise in improving physical fitness in (physically inactive) cancer patients.

### Psychosocial outcomes

This review found that yoga has large beneficial effects on distress, anxiety and depression, moderate beneficial effects on fatigue, general HRQoL, emotional function and social function, and a small and insignificant effect on sleep. There was insufficient evidence for effects on psychosocial outcomes that were studied less frequently including cognitive function, vigor, anger-hostility, spirituality, relaxation and mental health. More studies evaluating the effects of yoga on these outcomes are needed before we can draw firm conclusions.

The finding that yoga improves QoL, and reduces distress and depression concurs with findings from previous reviews and meta-analysis of yoga interventions for cancer patients and survivors [35,37]. In contrast, the current meta-analysis could not confirm previous findings on reductions in sleep disturbances in patients with breast cancer. Fatigue is among the most frequently occurring and debilitating complaints associated with cancer and cancer treatments [5,64]. Therefore, it is important to find effective strategies to reduce fatigue in cancer patients. In contrast to the meta-analysis of Lin et al. [37], we found a moderate significant effect size on fatigue. This is in line with a recent study of Bower et al. [58] who showed beneficial effects after 12 weeks of yoga classes on persistent fatigue in breast cancer survivors. In addition, patients themselves also perceived improvements in QoL, fatigue, stress, anxiety and depression [63]. The moderate-to-large effect sizes on these psychosocial outcomes seem larger than the small to moderate effect sizes of exercise [12,16,65-67] or psychosocial interventions [12,66,68]. However, our results have to be interpreted with caution due to small sample sizes in most studies. Furthermore effect sizes may also be influenced by patient selection, i.e. including also non-fatigued or non-depressed patients. Therefore, future studies should obtain insight in the most effective interventions to improve psychosocial outcomes. The current meta-analysis showed that yoga may be such an intervention.

### Methodological quality of studies

This review included a quality rating, and only one paper was of low quality. A major concern regarding the methodological quality of most included studies was that not all participants completed the yoga program and data were not analysed on an intention-to-treat basis. This may have introduced bias, overestimating the benefits of yoga. Only half of the studies reported class attendance, of which four studies indicated that intervention adherence was positively associated with some outcomes [32,53,54,57]. Whether adherence to the yoga sessions was affected by cancer-related symptoms or side-effects of cancer treatment was not reported.

Most studies separately reported the descriptive results of the outcomes for the yoga and control groups, and presented only p-values for the group differences. Many studies however, did not report effect sizes or other point estimates of the between-group differences, and their confidence intervals. Therefore we calculated standardized mean differences using means and standard deviations of the post-test values.

Furthermore, as with all exercise interventions, blinding was difficult. Because the control group usually consisted of either wait-list or usual care, participants were not blinded to the intervention, possibly introducing bias.

### Strengths and limitations

The extensive search in ten databases, the inclusion of RCTs, the methodological quality assessment and conduction of a meta-analysis are strengths of the study. Further, by only including studies focusing on yoga interventions that contained physical postures (asanas), we attempted to reduce the variability between the yoga interventions, thereby increasing the comparability of studies. Nevertheless, there may still remain some variability between the different types of yoga interventions included in this review. This may be reflected by the high heterogeneity. Other sources of high heterogeneity may be differences in instruments used to define the outcome, differences in patient groups (i.e. different stage of cancer, or different timing of the intervention with respect to primary cancer treatment), or differences in control groups. Because of the small number of studies, we were unable to conduct subgroup analyses to further reduce heterogeneity. Although we used random effects modelling to take into account the large heterogeneity, overall effect sizes should be interpreted with caution as they may vary somewhat among subgroups.

In general, publication bias endangers the external validity of reviews and meta-analyses. Also in this study, publication bias cannot be ruled out. Another limitation is the small sample size of some studies. In addition, some studies were conducted by the same research group, and other studies had multiple outcomes, increasing the probability of type 1 errors. Furthermore, most studies offered yoga to people based on having cancer, not based on having physical or psychosocial problems, which may have resulted in an underestimation of the beneficial effects of yoga.

The yoga interventions were conducted during various forms of cancer treatment; some were conducted post-treatment, and some studies included a mixed sample of patients during and post-treatment. Due to the limited number of studies, it is difficult to draw conclusions on the optimal timing for yoga interventions. Future studies should consider this issue.

Further, although effect sizes for many psychosocial outcomes were generally moderate to large, effect sizes of yoga interventions on physical function and functional well-being were small. This may indicate that the effect is small, or that some patients may have physical benefits from yoga whereas others may not, which may be indicative of the heterogeneity of cancer patients. Future studies with large sample sizes should identify moderators of the effect of yoga on physical and psychosocial outcomes in order to identify subgroups of patients whom may specifically benefit from yoga.

Finally, this review only included papers published in the English language. Although our searches were not limited to language, we may have missed important findings from yoga in Asia, in which practicing yoga is much more common than in Western countries. Nevertheless, this review included six studies that were conducted in Asia.

### Clinical implications

The emerging literature provides preliminary support for the feasibility and efficacy of yoga interventions for cancer patients. Only one adverse effect was reported in five studies that assessed adverse events, and the results indicate that yoga may improve physical well-being and psychosocial outcomes. Although the literature suggests yoga may be effective in improving physical outcomes in other patients and healthy elderly [29,30,34], evidence in cancer patients and survivors is generally insufficient to draw firm conclusions at this stage. In contrast, physical exercise has been shown to be effective in improving physical function and fitness in cancer patients and survivors [3,4,10,16]. However, for cancer patients and survivors who are unable or unwilling to participate in traditional aerobic or resistance exercise programs yoga may be an appropriate form of exercise [63] as it is especially suitable for those who perceive barriers to other forms of exercise [69]. Breast cancer patients have been found to perceive more barriers to exercise than age-matched controls, and higher barriers were associated with less exercise [70]. In a recent pilot study, breast cancer survivors reported minimal barriers and high motivation for participating in a yoga program [63]. All participants reported that yoga was beneficial and enjoyable, that they were confident that they could do the exercises, and that they were motivated to attend all classes [63]. However, evidence for yoga as an effective intervention to improve physical function and fitness is lacking and should be established by future studies. Future studies should also systematically assess and report adverse events related to yoga.

## Conclusion

This systematic review and meta-analysis of RCTs showed that yoga has strong beneficial effects on distress, anxiety and depression, moderate effects on fatigue, general HRQoL, emotional function and social function, small effects on functional well-being, and no significant effects on physical function and sleep disturbances. Results of the current review must be interpreted with caution due to the relative small sample sizes of most of the included studies. RCTs with larger sample sizes are needed to improve our understanding of the physical and psychosocial effects of yoga. Future studies should also address the optimal duration and frequency of yoga, the effects in patients with types of cancer other than breast cancer, and the optimal time point in the cancer and cancer treatment or rehabilitation trajectories for offering yoga interventions [63].

## Competing interests

The authors declare that they have no competing interests.

## Authors’ contributions

LB, JvU and MC have made substantial contributions to conception and design of the manuscript. LB and JvU have screened papers and conducted the quality rating and meta-analysis. IR has conducted the literature search. LB and JvU have been involved in drafting the manuscript. MC, IR, WB, JB, WvM have been involved in critically revising the manuscript. All authors read and approved the final manuscript.

## Pre-publication history

The pre-publication history for this paper can be accessed here:

http://www.biomedcentral.com/1471-2407/12/559/prepub
